# Ag–Ce_0.9_Gd_0.1_O_2−δ_-Based Nanocomposite Thin Film Air Electrodes for Low-Temperature Solid Oxide Cells

**DOI:** 10.1021/acsaem.4c02899

**Published:** 2025-02-27

**Authors:** Ozden Celikbilek, Matthew P. Wells, Judith L. MacManus-Driscoll, Gwilherm Kerherve, Laetitia Rapenne, David Muñoz-Rojas, Mónica Burriel, Marlu Cesar Steil, Elisabeth Siebert, Stephen J. Skinner

**Affiliations:** † Department of Materials, Imperial College London, Exhibition Road, London SW7 2AZ, U.K.; ‡ Institute of Engineering, Univ. Grenoble Alpes, CNRS, Grenoble INP, LMGP, Grenoble 38000, France; § Department of Materials Science and Metallurgy, University of Cambridge, Cambridge CB3 0FS, U.K.; ∥ Univ. Grenoble Alpes, Univ. Savoie Mont Blanc, CNRS, Grenoble INP, LEPMI, Grenoble 38000, France

**Keywords:** nanocomposite heterostructures, SOCs, oxygen electrodes, low-temperature SOCs, Ag-CGO, silver, PLD, thin films

## Abstract

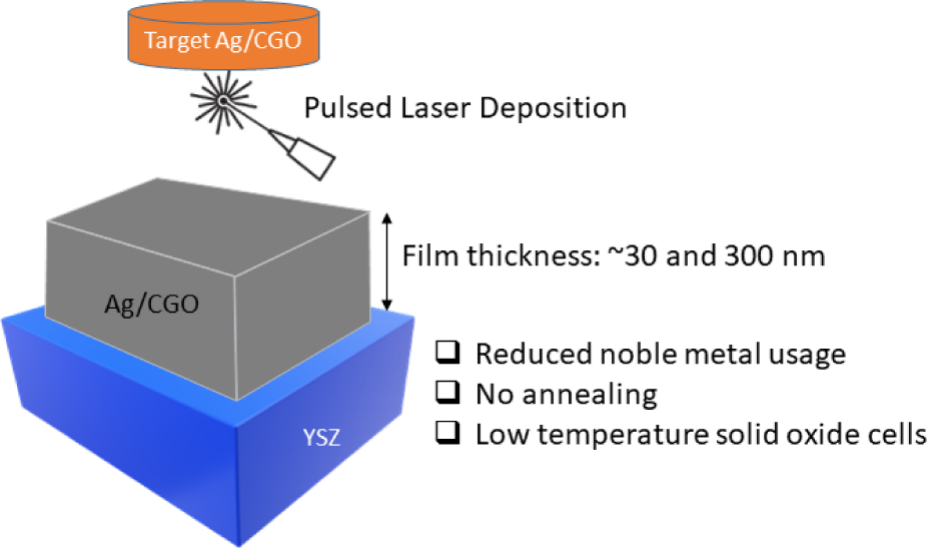

Understanding and controlling the interfaces between different materials is crucial for developing solid oxide cells (SOCs) with both high performance and durability for low-temperature operation (<700 °C). Current research focuses on evaluating microstructural designs and composite material interactions to optimize SOC performance. Nanocomposite heterostructures exhibit unique properties at the interfaces, which are achieved through precise control of the composition, thickness, and surface chemistry. In this investigation, our goal was to develop nanocomposite films using a combination of a metal and a metal oxide. Specifically, we successfully fabricated Ag–Ce_0.9_Gd_0.1_O_2−δ_ (Ag-CGO) nanocomposite thin films using pulsed laser deposition (PLD) in a single step. Dense Ag-CGO films with thicknesses of approximately 30 and 300 nm were grown on (100)-oriented yttria-stabilized zirconia (YSZ) substrates. The 300 nm–thick films exhibited an area-specific resistance (ASR) value of 22.6 Ω cm^2^ at 480 °C in a symmetrical cell configuration. This value is comparable to that of a micrometer scale–thick Ag electrode with a coarse porous microstructure. Therefore, Ag-CGO films represent a promising alternative to bulk Ag-based SOC electrodes by significantly reducing noble metal usage. The process described is suitable for integration into thin-film solid oxide fuel cell fabrication processes, as it eliminates the subsequent annealing step required to form a stable and active layer. Overall, this study provides valuable insights into enhancing the performance of metal/metal oxide thin films as SOC electrodes for low-temperature operation. While further investigations are necessary to optimize long-term stability, these films may also prove attractive for alternative catalytic applications operating at lower or ambient temperatures.

## 1 Introduction

SOCs have emerged as promising candidates for efficient and environmentally friendly devices for energy conversion. Through careful design, they can operate effectively over a wide temperature range. Low-temperature SOCs (LT-SOCs) operating at temperatures below 700 °C offer several advantages over intermediate- to high-temperature solid oxide cells (IT-SOCs and HT-SOCs). These advantages include increased durability, faster start-up, reduced thermal management requirements, compatibility with a wider range of fuels, and the potential for lower costs.^1^ However, there are still technical challenges at low temperatures due to reduced electrochemical performance and electronic conductivity of the state-of-the-art perovskite oxide materials.^2^ Microstructural design and material selection are critical in determining the overall performance and stability of the LT-SOCs. It is essential to address these challenges in order to fully unlock the potential of this innovative technology.

One intriguing approach to advancing LT-SOC technology is the utilization of nanocomposite heterostructures, which offer a unique opportunity to enhance the efficiency and durability of LT-SOCs, allowing tailored combinations of materials and optimizing their synergistic effects.^3−5^ Various combinations of perovskite and fluorite composites have been investigated for SOC applications and have shown improved oxygen reaction rate,^4,6−8^ enhanced ionic conductivity,^3,9^ and long-term material stability compared to their single-phase counterparts.^7,10^

Another approach to address performance and durability issues for LT-SOCs is through careful material selection. Noble metals such as platinum, silver, and gold have better catalytic performance and electronic conductivity at lower temperatures than perovskite ceramic oxides.^11^ Among these metals, silver has emerged as a particularly cost-effective option, with its price consistently remaining considerably cheaper than that of platinum and gold over the past two decades.

First-principles calculations suggest that oxygen reduction reactions occur more easily at the triple phase boundary (TPB) where the electronically conductive silver meets the gas phase and the ionically conductive electrolyte.^12^ To optimize performance, researchers have focused on extending these TPB sites throughout a larger electrode volume.^13^ A common strategy involves creating a composite electrode incorporating an ionically conductive material such as doped zirconia or doped ceria.^14−16^ For instance, ∼25 μm–thick Ag-CGO composites have been demonstrated as promising symmetrical electrode materials for reversible SOC applications, exhibiting superior performance as both oxygen and hydrogen electrodes compared to the state-of-the-art LSCF-CGO and Ni-YSZ, respectively.^17^ However, silver presents challenges due to the propensity of atomic oxygen to dissolve in the silver, particularly with its mobility increasing significantly above 650 °C.^18^ Consequently, high-temperature operations in SOCs pose a risk of strain in the silver lattice, which can lead to the formation of cracks.^19^ Furthermore, silver is susceptible to dewetting, island formation, and evaporation, even below 500 °C.^20,21^

Some studies have demonstrated that silver thin films can withstand temperatures up to 450 °C through a controlled balance between grain size and film thickness.^21,22^ Additionally, previous research has explored coating of the surface of silver thin films with ionically conducting nanoparticles.^13,14,21−23^ This approach prevents thermal agglomeration and enhances the TPB.

Several deposition techniques like infiltration,^24^ ALD,^25,26^ CVD,^27^ and sputtering^14,23^ can create composite silver thin films capped with ionic conductors. However, these methods often suffer from slow deposition rates, long and complex procedures, or high-temperature treatments for crystallization.

This study examines for the first time the feasibility of depositing a composite thin film containing metallic silver (Ag) and gadolinium-doped ceria (CGO) in a single step by using pulsed layer deposition (PLD), eliminating the need for subsequent high-temperature annealing. Metallic silver is expected to provide high electrical conductivity, while CGO is anticipated to mitigate the thermal agglomeration of silver and enhance oxygen reaction kinetics through its good ionic conductivity. Importantly, the elimination of the subsequent annealing step to form a stable and active layer renders this process suitable for integration into thin-film fabrication processes. The stability of the films was studied at two different thicknesses, approximately 30 and 300 nm, grown on (100)-oriented yttria-stabilized zirconia (YSZ) single crystal substrates. The microstructure, morphology, crystal structure, and surface chemistry were assessed by using various characterization techniques to enable a comparison of the electrochemical performance of the nanocomposite films. The techniques used in this study include scanning electron microscopy (SEM), atomic force microscopy (AFM), transmission electron microscopy (TEM), X-ray diffraction (XRD), secondary ion mass spectrometry (SIMS), X-ray photoelectron spectroscopy (XPS), and electrochemical impedance spectroscopy (EIS). The practical use of these films was also compared to a micrometer scale–thick Ag electrode that was paint-brushed on the YSZ substrates. Through these measurements, we find that the novel Ag-CGO thin-film air electrodes presented herein exhibit good initial performance, with an ASR value of 22.6 Ω cm^2^ at 480 °C. Therefore, by demonstrating electrochemical performance comparable to bulk Ag-based oxygen electrode materials despite a significantly reduced Ag content and a simple fabrication procedure, the present work demonstrates a promising route toward the design of low-temperature SOCs, although further work is needed to address long-term stability for SOC applications and to explore potential use in other catalytic processes.

## 2 Methods

### 2.1 Thin Film Deposition

Films were grown by pulsed laser deposition (PLD) on (100)-oriented YSZ single crystal substrates (10 × 10 × 0.5 mm, Crystec GmbH). The Ag-CGO targets for the PLD growth were made by mixing 70:30 wt % Ag metal (Merck, ≥99.9% purity, particle size 2–3.5 μm) and Ce_0.9_Gd_0.1_O_2–δ_ powders (Praxair, 99.9% purity, surface area: 6.4 m^2^ g^–1^) in a rotary ball mill for ∼12 h. The composite was pelletized into 30 mm diameter discs using a uniaxial press, followed by consolidation in an isostatic press (350 MPa) and sintering at 900 °C for 2 h in air.

The films were deposited with a 248 nm KrF excimer laser (Lambda Physik, Inc.) with a 25 ns pulse duration. During the experiments, the laser fluence was approximately 0.8 J/cm^2^, while the laser repetition rate was 10 Hz. The target–substrate distance was set to 4.5 cm. The substrate temperature was set to 250 °C, and the pressure was set at 5 × 10^–6^ Torr. The number of pulses was 4800 to get the 30 nm–thick films and was 24,000 to get the 300 nm–thick films.

### 2.2 Structural Characterization

X-ray diffraction (XRD) measurements for samples on single-crystal substrates were conducted by using a Panalytical Empyrean high-resolution X-ray diffractometer using Cu Kα radiation (λ = 1.5405 Å). The data were collected in the θ/2θ geometry with a scan range between 10° and 80° in 2θ and a 0.02° step width with a 1 s step time.

### 2.3 Microstructure and Morphology Characterization

The microstructure of the films was studied using a field-emission gun scanning electron microscope (FEG-SEM Zeiss Gemini 300, Carl Zeiss Microscopy GmbH, Oberkochen, Germany) operating at an accelerating voltage of 3 kV using an In-lens detector and a ∼5 mm working distance. Atomic force microscopy (AFM) measurements were performed on films by using a Bruker Multimode 8 system in tapping mode. Commercial silicon cantilevers (Budget Sensors Ltd.) with a resonance frequency of 300 kHz and a spring constant of 40 N/m were used to image 0.5 μm^2^ areas at a scan frequency of 1 Hz. AFM data were analyzed using open-source software Gwyddion 2.53.^28^ Transmission electron microscopy (TEM) images were carried out at 200 kV with a JEOL 2010 microscope (with a resolution of ∼0.19 nm) for TEM and HRTEM and with a JEOL 2100F for STEM EDX analysis. Cross-sectioned samples were prepared by automated polishing, using the MultiPrep system (Allied High Tech Products, Inc.). The final polishing was performed using a felt-covered disc impregnated with a silica solution until the appearance of the first extinction fringe among those of equal thickness. Ar-ion milling was then used to minimize the total thickness.

### 2.4 Electrochemical Characterization

For electrochemical characterizations, double-side polished YSZ substrates were used to deposit films symmetrically on both sides. 80 nm–thick and dense Au layer was sputtered on both sides for current collection on top of the Ag-CGO thin films. For electrochemical comparison, Ag electrodes were brush-painted on YSZ electrodes and fired at 800 °C for 2 h in air. The electrochemical characterization of the films was performed using a Solartron 1260 frequency response analyzer over the temperature range of 300–480 °C and in the frequency range of 13 MHz to 0.01 Hz at an amplitude of 100 mV. All symmetrical samples were measured at the open-circuit voltage. The measurements were taken only on heating, and measurements were terminated at 480 °C. A dwell time of 1 h was set between the measurements. The symmetrical samples were contacted with Au grids (Goodfellow, 1500 wire/in.) and then sandwiched between Al_2_O_3_ blocks, which were pressed to ensure maximum contact points. The impedance diagrams were fitted with electrical equivalent circuits using ZView 2 software (3.5f, Scribner Associates). The resistance multiplied by the geometric surface area of the electrodes and divided by 2 gave the area-specific polarization resistance.

### 2.5 Surface Characterization

The surface chemistry and electronic structure were characterized by using a Thermo Fisher Scientific K-Alpha+ XPS system operating at 2 × 10^–9^ mbar base pressure at ambient temperature. The system incorporates a 180° double-focusing hemispherical analyzer with a 128-channel detector, while the Al Kα X-ray source generates a 6 mA emission current with a spot size of 400 μm^2^. Pass energies of 200 and 20 eV were used for the survey and core-level spectra, respectively. The quantitative analyses were performed using Avantage software.^29^ A Shirley background was subtracted from the data, and peaks were fitted by using a convolution of Gaussian and Lorentzian peak shapes. Ag 3d, Ce 3d, Gd 3d, O 1s, and C 1s spectra were analyzed. The binding energies were corrected to the C 1s peak position at 284.8 eV, originating from the surface hydrocarbons.

Secondary ion depth profiles in the films were analyzed using time-of-flight SIMS (ToF-SIMS) on a ToF-SIMS5 machine (IONTOF GmbH, Münster, Germany) equipped with a bismuth liquid metal ion gun. A 25 keV Bi^+^ primary ion beam was used to generate secondary ions in the high current bunch mode, and 10 keV argon cluster beam (Ar_n_^+^) was used for depth profiling. Positive secondary ion species originating from the film and substrate were monitored. The analysis area was 100 × 100 μm^2^, and the sputtering area was 300 × 300 μm^2^.

## 3 Results and Discussion

**Figure 1:**
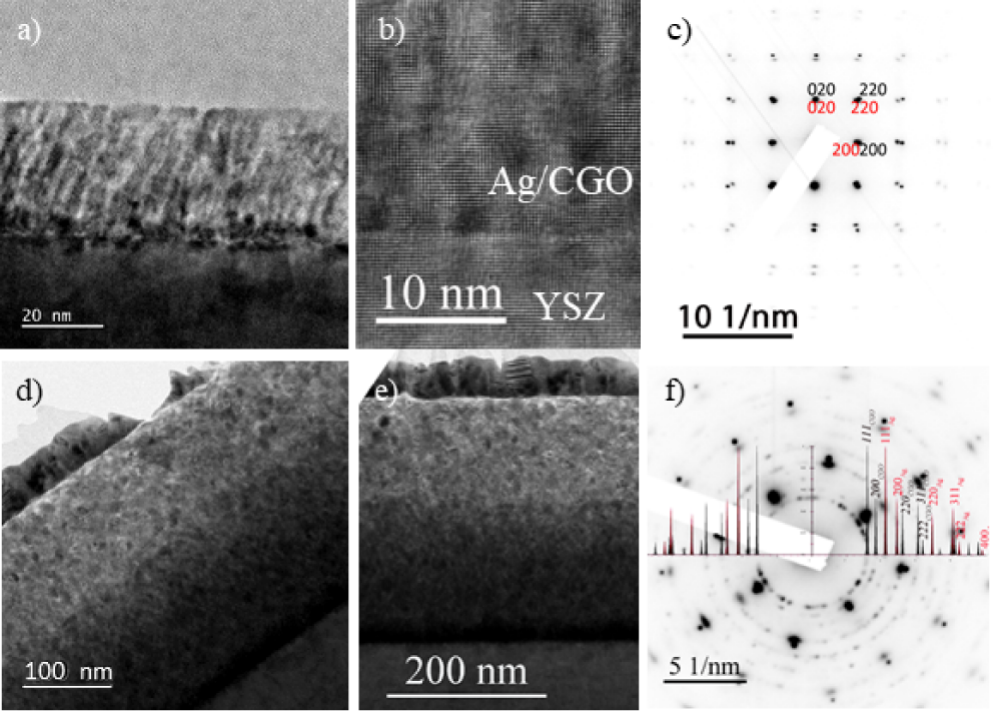
Cross-sectional bright field TEM micrographs of (a, b) 30 nm–thick and (d, e) 300 nm–thick Ag-CGO films deposited on the YSZ (100) substrate. Selected area electron diffraction (SAED) patterns are taken on images b and e are shown next to them in images c and f, respectively. The SAED pattern in image c is taken at the Ag-CGO interface of the 30 nm film in image b along the [100] zone axis, showing diffraction planes of CGO (red) and YSZ (black). The SAED pattern shown in image f is taken on the 300 nm–thick film shown in image e, indicating diffraction planes of Ag (red) and CGO (black).

TEM analysis was conducted to examine various aspects of the film, including thickness, grain size, orientation, crystallinity, and phase contrast (Figure 1). Cross-section images in Figure 1a,d show films with approximate thicknesses of 30 and 300 nm, respectively, deposited on a single crystal YSZ (100) substrate. The surfaces of both lamellae were coated with 1 nm Au/Pd nanoparticles to prevent electron beam charging, albeit resulting in a reduced high-resolution imaging quality. The microstructure of the 30 nm–thick film appears to show an ordered growth with nanocolumnar elongated grains (Figure 1a,b), while the 300 nm thick film displays more randomly dispersed grains (Figure 1d,e). Both micrographs reveal the highly crystalline nature of the films. Selected area electron diffraction (SAED) patterns were then recorded on the images shown in Figure 1b,e. The SAED pattern of the 30 nm film (Figure 1c) indicates epitaxial growth of CGO on the (100)-oriented YSZ substrate, whereas the diffraction pattern of Ag was not discernible due to the small quantity of Ag present compared to the CGO in the analyzed area. Conversely, the SAED pattern of the top part of the 300 nm–thick film (Figure 1f) suggests polycrystalline growth on YSZ. Notably, at the bottom interface of the 300 nm–thick film, the SAED pattern reveals that the few first nanometers of the film grow with an epitaxial relationship between the CGO and YSZ grains (Figure S1).

It is important to note that the deposition rates of metals by pulsed laser deposition are typically lower than of ceramic materials.^30^ To compensate for the slower sputtering rate of silver, we adjusted the PLD target composition to be Ag-rich (70%). While it is not possible to directly quantify the amount of silver transferred to the film, elemental distribution across the films was examined using STEM-EDX. For the 30 nm film, STEM-EDX mapping (Figure S2) revealed that both Ag and Ce/Gd are present throughout the entire film thickness. Similarly, for the 300 nm film, multiple point analyses along the film thickness confirmed a homogeneous distribution of Ag and Ce/Gd across the entire thickness (Figure S3 and Table S1). This observation supports the conclusion that the deposition method ensures silver is distributed across the entire film thickness.

**Figure 2:**
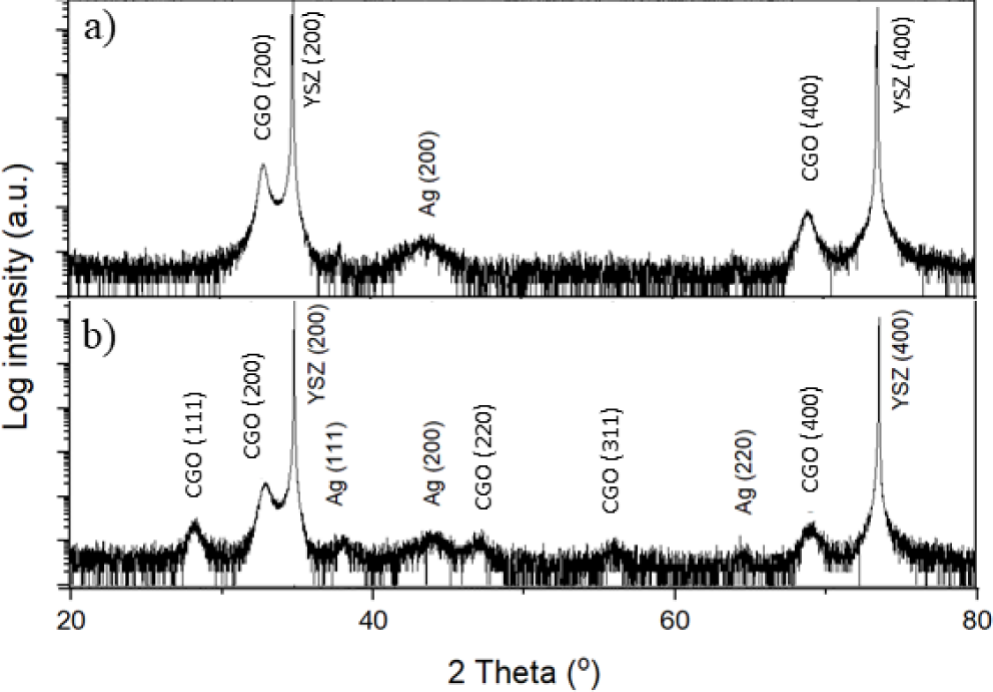
XRD diffractograms of (a) 30 and (b) 300 nm–thick Ag-CGO film deposited on YSZ (100) single crystal substrates.

X-ray diffraction (XRD) analysis (Figure 2) reveals the crystal structure and orientation of Ag-CGO thin films grown on YSZ (100) substrates. The diffractograms show peaks from both the films and the substrate. For the 30 nm–thick film (Figure 2a), only reflections corresponding to the (h00) planes of Ag and CGO are observed along with those of the YSZ substrate. This indicates highly oriented growth of the film, aligning its crystal planes with the substrate. Conversely, the pattern of the 300 nm–thick film (Figure 2b) exhibits peaks characteristic of the cubic fluorite structure for both Ag and CGO, confirming polycrystalline growth as observed in the SAED patterns (Figure 1f and Figure S4). Notably, the CGO peaks are well-defined, indicating a high crystallinity. However, the Ag peaks are weaker in intensity and broader than the CGO. This suggests a broad particle size distribution of silver within the film. Overall, these results indicate the biphasic nature of these films.

**Figure 3:**
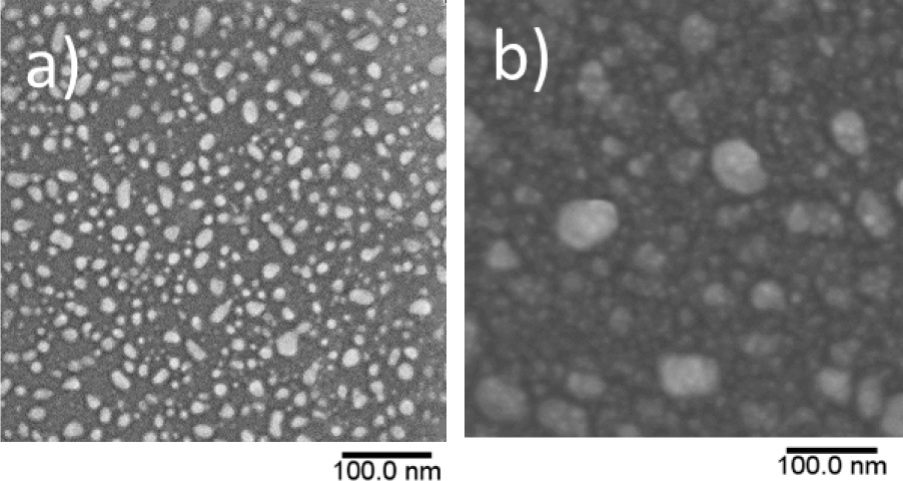
Scanning electron micrograph of (a) 30 nm and (b) 300 nm–thick films grown on YSZ (100) showing phase contrast between Ag and CGO.

The morphology and surface topography of the films were investigated by SEM (Figure 3). The images show clear phase contrast with light and dark gray colors. Notably, the light gray grains, associated with Ag, appear to agglomerate, resulting in an uneven particle size distribution at the surface. This observation correlates with the low intensity and broad XRD peaks observed for Ag in Figure 2. Atomic force microscopy (AFM) was used to further investigate the surface topography using height and phase images (Figure S5). The grain size of the 30 nm–thick film is 7 ± 3 nm, and that of the 300 nm–thick film is 50 ± 15 nm, representing a substantial difference between the films. The root-mean-square (rms) roughness of the 30 nm–thick film is calculated as 0.38 ± 0.1 nm and that of 300 nm–thick films is calculated as 4.47 ± 0.06 nm.

**Figure 4:**
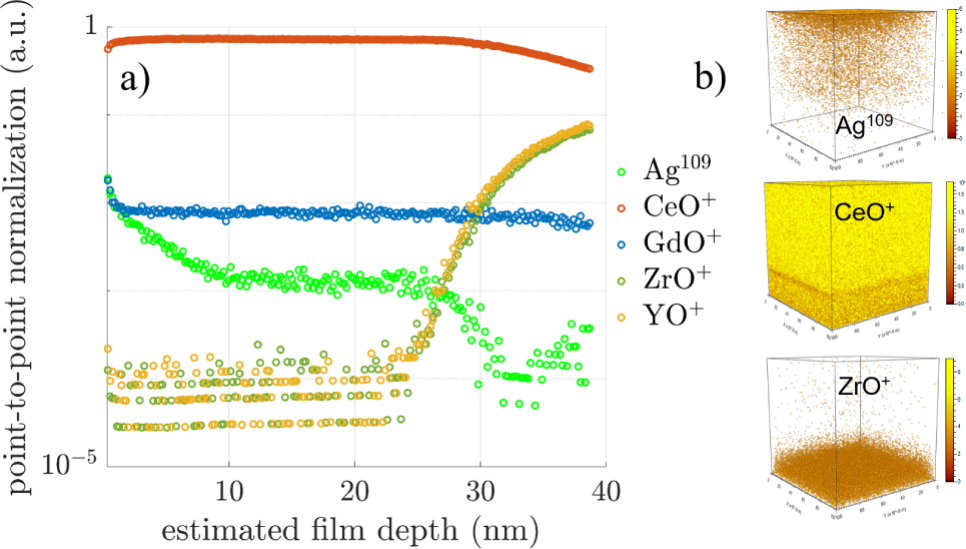
(a) Positive SIMS depth profile analysis of the 30 nm–thick Ag-CGO film. The *y*-axis corresponds to the point-to-point normalization of the total counts intensity on the logarithmic scale. The onset of the film/substrate interfacial region is estimated to be around the 30 nm film depth. (b) 3D rendering of Ag-109 and CeO^+^ in the film and ZrO^+^ in the substrate.

While STEM-EDX maps provided evidence of silver distribution across the 30 nm film thickness, ToF-SIMS was employed to complement it with its enhanced depth profiling capabilities and the ability to analyze a larger area, providing a more comprehensive understanding of the compositional distribution (Figure 4). Positive secondary ion species originating from both the film and the substrate were monitored. The positive SIMS depth profiles (Figure 4a) display compositional variations from the film surface to the substrate. Notably, for CGO, a slightly Gd-rich and Ce-deficient layer is visible at the surface, with their signals plateauing below the surface until reaching the substrate. On the other hand, a significantly higher Ag intensity is observed at the surface, decreasing continuously until reaching about 10 nm deep into the film, where it stabilizes (Figure 4a,b).

**Figure 5:**
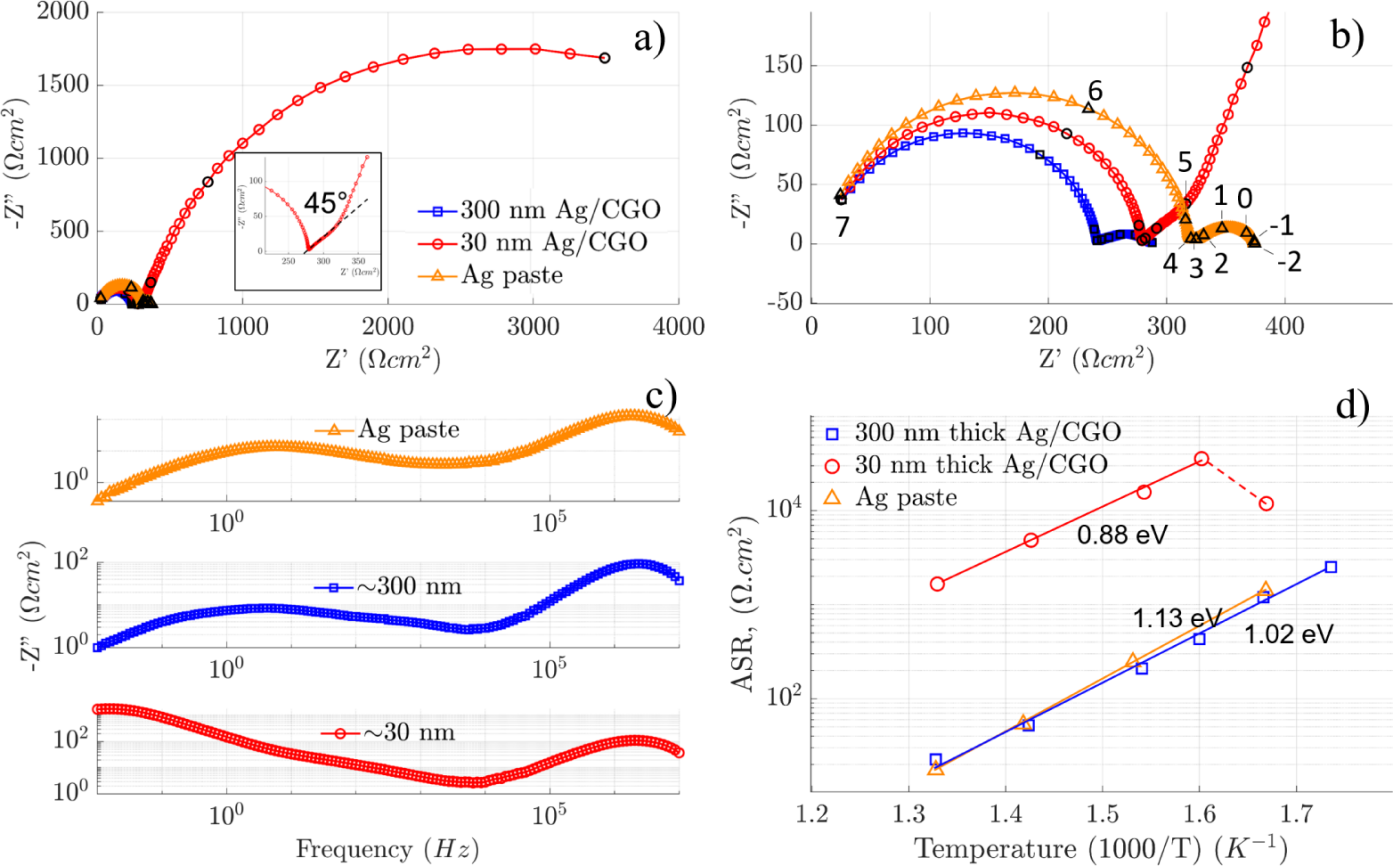
(a) Nyquist plot of the three films (30 and 300 nm–thick films and Ag paste) measured at 428 °C in OCV and synthetic air. The inset shows a magnified view of the 30 nm film response, highlighting the characteristic impedance behavior with a 45° angle and a semicircle. (b) Magnified view of the Nyquist plot of panel a. Black circles and numbers (logarithmic values) represent a decade change in frequency range (10^–2^ to 10^7^ Hz). (c) Bode plot of the impedance plots shown in panel a. (d) Arrhenius plot showing the area specific resistance (ASR) as a function of temperature for 30 and 300 nm–thick films deposited on YSZ (100) substrates. The response of Ag paste prepared by brush painting is included for comparison.

The oxygen reaction response at equilibrium and the stability of the films were assessed by electrochemical impedance spectroscopy. Representative Nyquist and Bode plots measured at 428 °C are illustrated in Figure 5a–c. The plots of the 300 nm film closely resemble those of the thick Ag electrode with a coarse porous microstructure, suggesting a similar oxygen exchange mechanism. Both exhibit two distinct arcs at the characteristic peak frequencies (*f*_c_) of around 2 MHz and 5 Hz. The plots of the 30 nm film also display a high-frequency arc with *f*_c_ = 2 MHz, but the low-frequency contribution is shifted toward a lower *f*_c_ at 0.08 Hz and it contributes most significantly to the ASR. The high-frequency semicircle with *f*_c_ = 2 MHz observed in all films has a capacitance value of around 10^–11^ F^31^ and is attributed to the resistance of the YSZ electrolyte (Figure S6). For both Ag-CGO films, a small shoulder is visible at around *f*_c_ = 10^3^ Hz, which can be attributed to the charge transfer at the Ag-CGO and/or Ag-YSZ interface.

The inset of Figure 5a highlights the distinct profile of the low-frequency response of the 30 nm film. It starts with a 45° line followed by a semicircle, resembling the impedance of a dense MIEC.^32,33^ On the other hand, the impedance of the 300 nm film (Figure 5b) resembles that of the thick and porous Ag electrode with a Gerischer profile.

The Arrhenius plot of the area-specific resistance (ASR, Ω cm^2^) shows the temperature dependence of the total electrode resistance measured at open circuit voltage between 300 and 480 °C on heating (Figure 5d). Several notable observations emerge from the analysis. A sharp increase in ASR is observed in the 30 nm–thick film on heating from 325 to 350 °C, possibly due to microstructural evolution. At 480 °C, an ASR value of 1658 Ω cm^2^ was measured. In contrast, the 300 nm film exhibits an order of magnitude enhancement in the ASR values within the same temperature range. Notably, a value of 22.6 Ω cm^2^ is recorded at 480 °C, comparable to a micrometer–thick Ag electrode with a coarse porous microstructure (Figure S7) with similar ASR and activation energy values. Compared to the state-of-the-art LSCF thin films with a similar thickness to our films, which report an ASR of 85 Ω cm^2^ at 450 °C^34^ or of approximately 70 Ω cm^2^ at 450 °C for LSCF/CGO thin films,^35^ the Ag/CGO nanocomposite offers a promising alternative.

**Figure 6:**
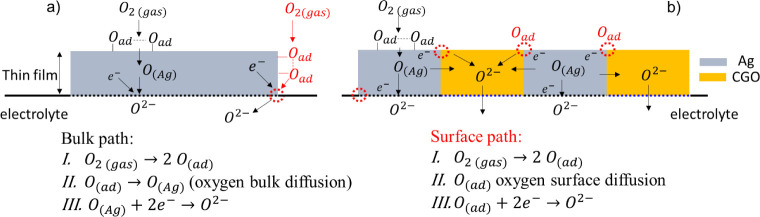
Simplified schematic illustration of possible oxygen exchange reaction mechanisms involving bulk and surface path in a (a) thin and dense Ag film and (b) thin and dense Ag-CGO electrode.

In terms of each individual process, the oxygen electrode reactions may involve a series of steps, including dissociative adsorption, diffusion (either surface or bulk), charge transfer at the TPB, and ionic transfer at the electrode/electrolyte interface.^36^ Two diffusion pathways have been reported in a silver electrode and can be illustrated as the bulk path and the surface path (Figure 6a). In the bulk path, oxygen from the gas phase adsorbs and incorporates onto the Ag surface, followed by diffusion of neutral oxygen species within the silver lattice.^37,38^ Subsequently, the charge transfer process to oxygen occurs at the Ag–electrolyte interface. The surface path also involves adsorption and incorporation of oxygen species on the surface, coupled with surface diffusion toward a TPB to be reduced. The charge transfer then occurs at the TPB at the Ag–electrolyte interface. The operating temperature appears to play a role in whether one path prevails over the other. At higher temperatures (700–900 °C), bulk diffusion of oxygen within the silver grains appears to control the reaction rate.^38^ At lower temperatures (300–500 °C), bulk diffusion was not identified as the rate-determining step for a 600 nm Ag film but rather the dissociative adsorption/reduction of oxygen.^39^

In a composite thin film of Ag with ionically conducting CGO, one can expect two possible scenarios, as illustrated in Figure 6b. The first involves diffusion of atomic oxygen in the Ag bulk and charge transfer at the Ag–YSZ interface. The second scenario involves surface diffusion of adsorbed oxygen species to the Ag–CGO interface, followed by charge transfer at that interface. This is then followed by bulk migration of oxide ions within CGO grains and charge transfer at the CGO–YSZ interface. The observed Gerischer impedance profile in this work likely arises from surface diffusion coupled with a slow surface exchange or a charge transfer process.^40^

After the electrochemical tests, further characterization of the 30 nm–thick film was not pursued, as visual inspection suggested a significant loss of Ag and the sputtered gold current collector layer. This is likely attributable to the high mobility of silver at the tested temperatures, leading to processes such as dewetting, island formation, and/or evaporation. Based on these observations, the 30 nm–thick Ag-CGO film is not suitable for low-temperature operation in SOCs. Conversely, the 300 nm film performs comparably to a much thicker silver electrode with a coarse, porous microstructure and merits further investigation for its long-term stability.

**Figure 7:**
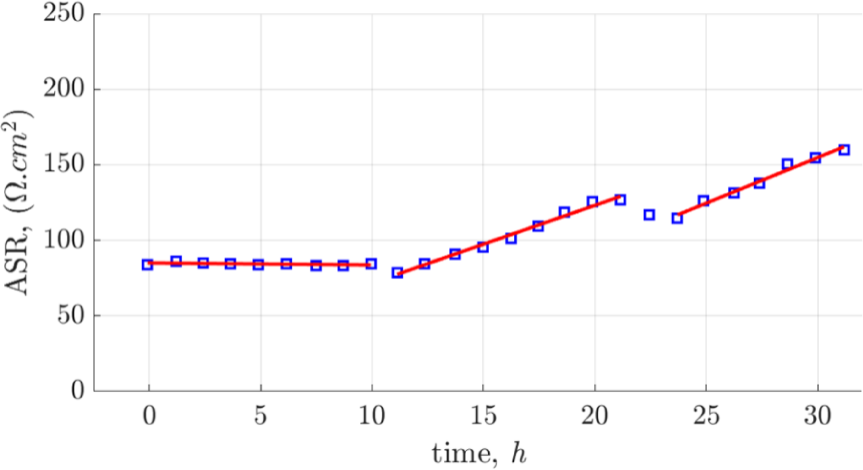
Stability test of the ∼300 nm–thick film at 371 °C for 31 h. The EIS measurements were taken at open circuit potential and under a flowing O_2_ atmosphere.

In the following, the stability of the 300 nm–thick Ag-CGO film deposited on the YSZ (100) substrate was evaluated on symmetrical cells. The measurements were conducted at a fixed temperature of 371 °C for approximately 31 h. Following a 1 h dwell period at the set temperature, impedance data were collected. Figure 7 shows the variation in the ASR value as a function of time. The initial 10 h exhibit a relatively stable profile. Afterward, a monotonic increase in ASR is observed with a rate of approximately 5% per hour, except for a brief decrease likely caused by minor temperature fluctuations within the setup.

**Figure 8:**
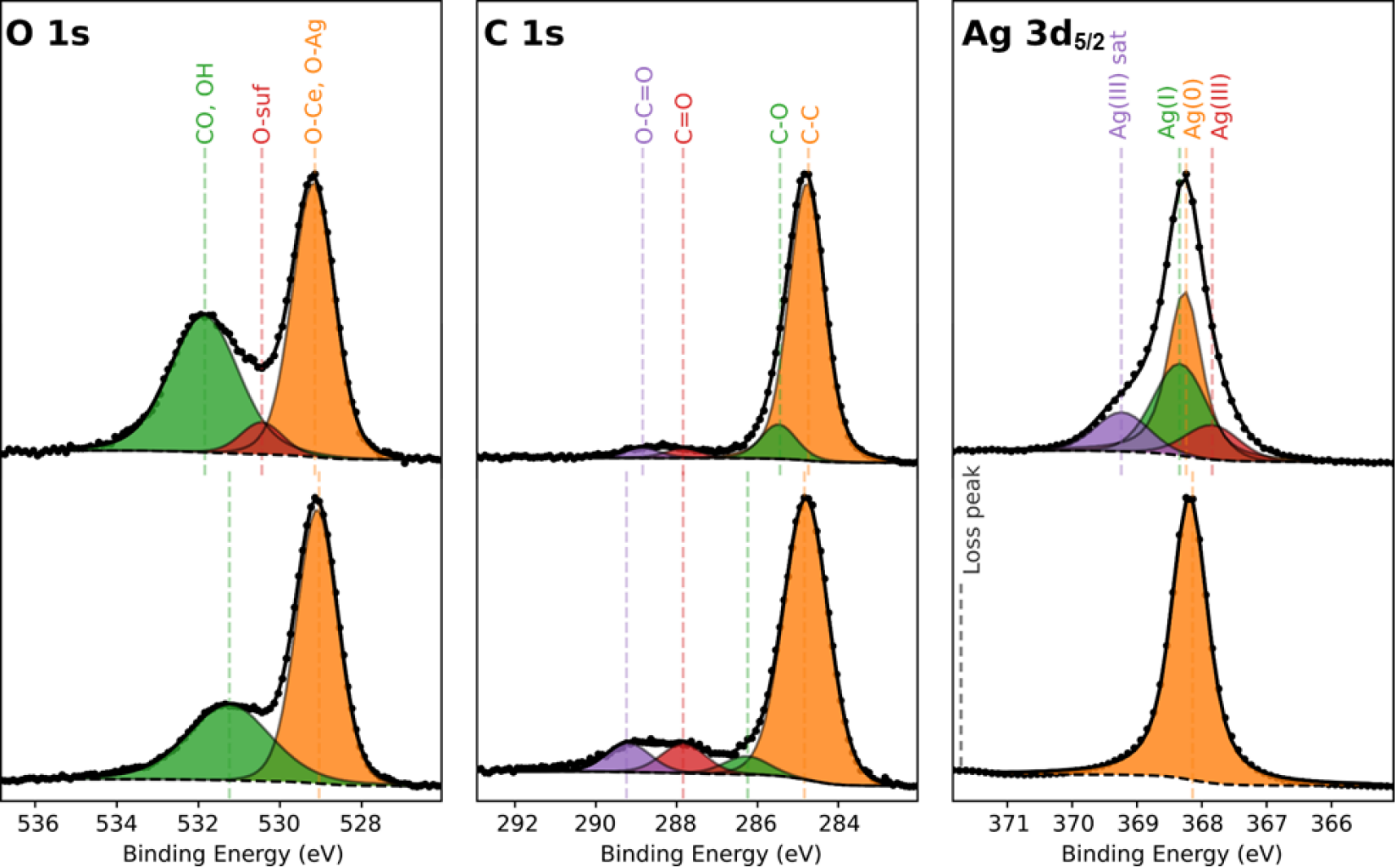
XPS core level spectra fittings of the 300 nm–thick films after (top images) and before (bottom images) the stability test.

Chemical and microstructural changes within the oxygen electrode can be considered as primary contributors to the observed degradation. To verify this, the surface composition and chemical states of the elements were analyzed via ex situ XPS measurements after stability tests (Figure S8). The dynamic interaction of oxygen with silver at high temperatures^41,42^ can lead to the oxidation of metallic silver at SOC operating temperatures. Figure 8 shows Ag 3d, C 1s, and O 1s core-level XPS spectra of an aged (top figures) and an as-deposited sample (bottom figures). O 1s and C 1s species showed different levels of adventitious carbon (284.8 eV) and other weakly adsorbed surface species like CO–, −OH, etc. The Ag 3d_5/2_ core-level spectrum before heat treatment shows a characteristic metallic silver peak at 368.3 eV, with an fwhm of 0.6 eV and an associated plasmon loss feature at around 371.8 eV. After heat treatment, the peak exhibits broadening at both lower and higher binding energies, which is attributed to the oxidation of Ag in the mixed Ag(I)/Ag(III) oxide form.^43^ Similar to Ferraria et al.,^43^ the Ag(I) oxide component appears at a binding energy close to one of the metallic peak. This differs from other values found in the literature^44^ and can be attributed to changes in the Madelung potential in a mixed oxide system. Based on the compositional analysis (Tables S2 and S3), approximately 43% of the initial metallic silver was oxidized to a combination of Ag(I) and Ag(III) oxides.^43^ The chemical composition analysis revealed a change in the ratio of Ag(total) to (Ce+Gd) for the 300 nm film. Before the stability test, the ratio was approximately 67.7:32.3, while it shifted to 58:42 after the test. This observation suggests a decrease in the relative concentration of silver, in particular, at or near the surface of the film. Further investigation of this sample using TEM (Figure S9) revealed a significant decrease in film thickness from the initial 300 nm to approximately 130 nm after stability tests, along with the disruption of the sputtered gold layer’s continuity. Top-view SEM images and EDX analysis of the film (Figure S10) demonstrate regions where the current collecting layer does not uniformly cover the film after stability tests, potentially contributing to the observed degradation in electrochemical performance. The significant thickness reduction of the 300 nm film after heat treatment likely arises from silver’s high mobility at elevated temperatures, leading to island formation and/or evaporation. Supporting this, Simrick et al. demonstrated that Ag thin films deposited on YSZ substrates undergo microstructural reorganization during thermal annealing (250–550 °C).^21^ Their findings suggest a dependence of these changes on the initial film thickness, annealing temperature, and duration. For instance, the Ag surface coverage of a 100 nm–thick film was 23% compared to a 77% coverage in the case of the 820 nm–thick film after annealing of both films at 550 °C for 1 h. Thicker films offer greater initial material volume, providing better resistance to such degradation, compared to thinner films. Similarly, Gilbert et al. studied Ag/YSZ and Ag/GDC thin film electrodes for catalytic applications, specifically ethylene epoxidation, at operating temperatures of around 220 °C. They observed Ag mobility at temperatures as low as 260 °C and proposed that sintering the films at higher temperatures compared to the operation temperature, such as 350 °C for 2 h, likely contributes to their thermal stability. This approach offers valuable insights into strategies for enhancing Ag electrode stability under operational conditions.^45^

In this study, we investigated the potential of silver electrodes combined with a CGO phase in a novel microstructure for low-temperature solid oxide cell applications. We have shown that our approach achieves comparable initial performance to thicker silver films but with significantly reduced silver content. This highlights the potential for material optimization and reduced silver usage in SOC electrodes. However, long-term stability remains a critical area for further research. While the ability to synthesize metal and metal oxide phases in a single step at a low temperature (250 °C) using PLD is a promising finding, further investigation is required to improve the durability of the material under realistic SOC operating conditions.

## 4 Conclusions

In this study, we have shown the feasibility of growing metal–metal oxide nanocomposite thin films in a single step using pulsed laser deposition. Ag-CGO films with thicknesses of approximately 30 and 300 nm were successfully grown on single crystal YSZ substrates. The 30 nm–thick film with an average grain size of 7 nm and that of 300 nm–thick film with an average grain size of 50 nm exhibited epitaxial growth, while the 300 nm film was polycrystalline. The 300 nm–thick film exhibited almost 2 orders of magnitude lower area-specific resistance value compared with the 30 nm–thick film. A value of 22.6 Ω cm^2^ was recorded at 480 °C comparable to a thick Ag electrode with a coarse porous microstructure. Notably, this was attained with considerably smaller film thickness and substantially reduced silver content. Nevertheless, the high degradation rate remains a challenge for long-term SOC operation. Despite that, these Ag-CGO films, with their low silver content and comparable performance to bulk Ag, hold the potential for alternative catalytic applications operating at lower or ambient temperatures, where silver instability is less of a concern.

## Supporting Information Available

Cross-sectional HRTEM micrographs, STEM-EDX elemental maps, AFM images, Arrhenius plot of YSZ conductivity, SEM images of brush-painted Ag electrode, XPS spectra of samples before and after the electrochemical stability tests (PDF)
