# Supplementary material for: Ag–Ce0.9Gd0.1O2−δ-Based Nanocomposite Thin Film Air Electrodes for Low-Temperature Solid Oxide Cells
Source: ACS Appl Energy Mater. 2025 Feb 27;8(5):2828–36. doi: 10.1021/acsaem.4c02899 (PMC11898068; doi:10.1021/acsaem.4c02899)
Supplement: Supplementary file 1 — ae4c02899_si_001.pdf [file ae4c02899_si_001.pdf]

Supporting Information

Ag-$Ce_{0.9}Gd_{0.1}O_{2-\delta}$ Based Nanocomposite Thin Film Air Electrodes for Low Temperature Solid Oxide Cells

Ozden Celikbilek (1,2) †, Matthew P. Wells (3), Judith L. MacManus-Driscoll (3), Gwilherm Kerherve (1), Laetitia Rapenne (2), David Muñoz-Rojas (2), Mónica Burriel (2), Marlu Cesar Steil (4), Elisabeth Siebert (4), Stephen J. Skinner (1)*

(1) Department of Materials, Imperial College London, Exhibition Road, London SW7 2AZ, United Kingdom

(2) Univ. Grenoble Alpes, CNRS, Grenoble INP[¥], LMGP, 38000 Grenoble, France

[¥] Institute of Engineering, Univ. Grenoble Alpes

(3) Department of Materials Science and Metallurgy, University of Cambridge, Cambridge CB3 0FS, United Kingdom

(4) Univ. Grenoble Alpes, Univ. Savoie Mont Blanc, CNRS, Grenoble INP, LEPMI, 38000, Grenoble, France

Corresponding Author

*ozden.celikbilek@cea.fr

Present Addresses

† Univ. Grenoble Alpes, CEA-Liten, DTNM, 38000 Grenoble, France

Keywords: Nanocomposite heterostructures, SOCs, oxygen electrodes, low-temperature SOCs, Ag-CGO, silver, PLD, thin films

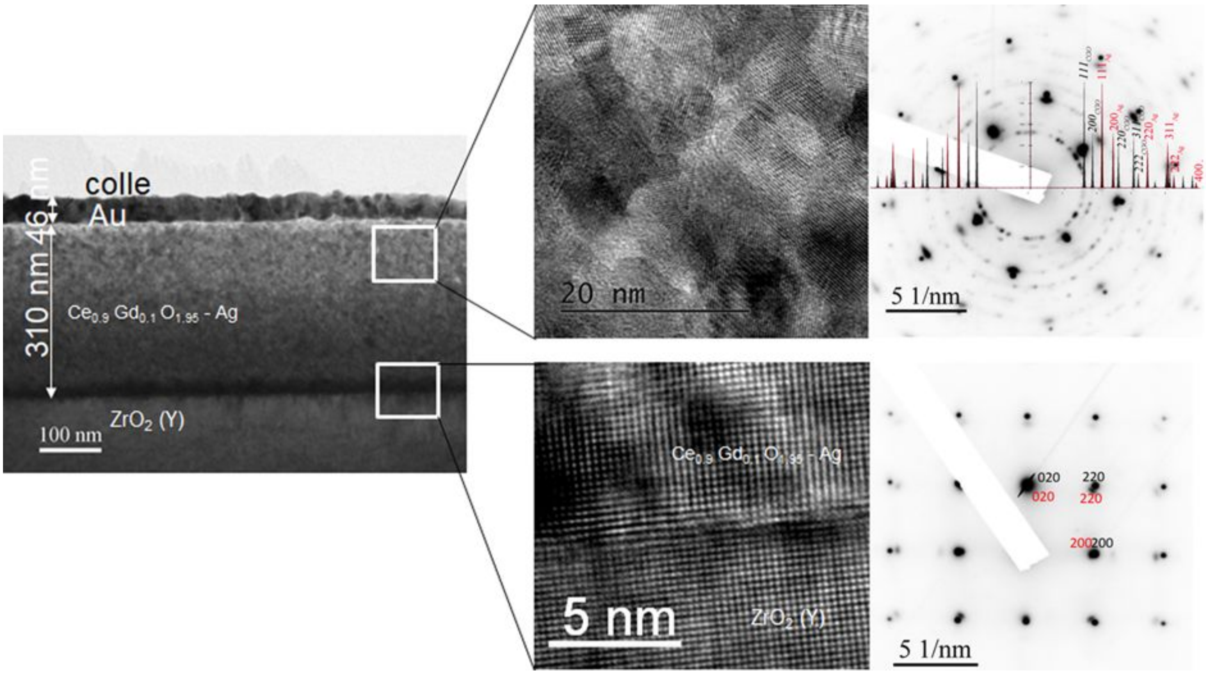

Fig. S1. a) Cross-sectional bright field HRTEM micrograph of 300 nm thick Ag-CGO film deposited on YSZ (100) substrate. The SAED pattern is taken at the Ag-CGO interface along the [100] zone axis, showing diffraction planes of CGO (red) and YSZ (black).

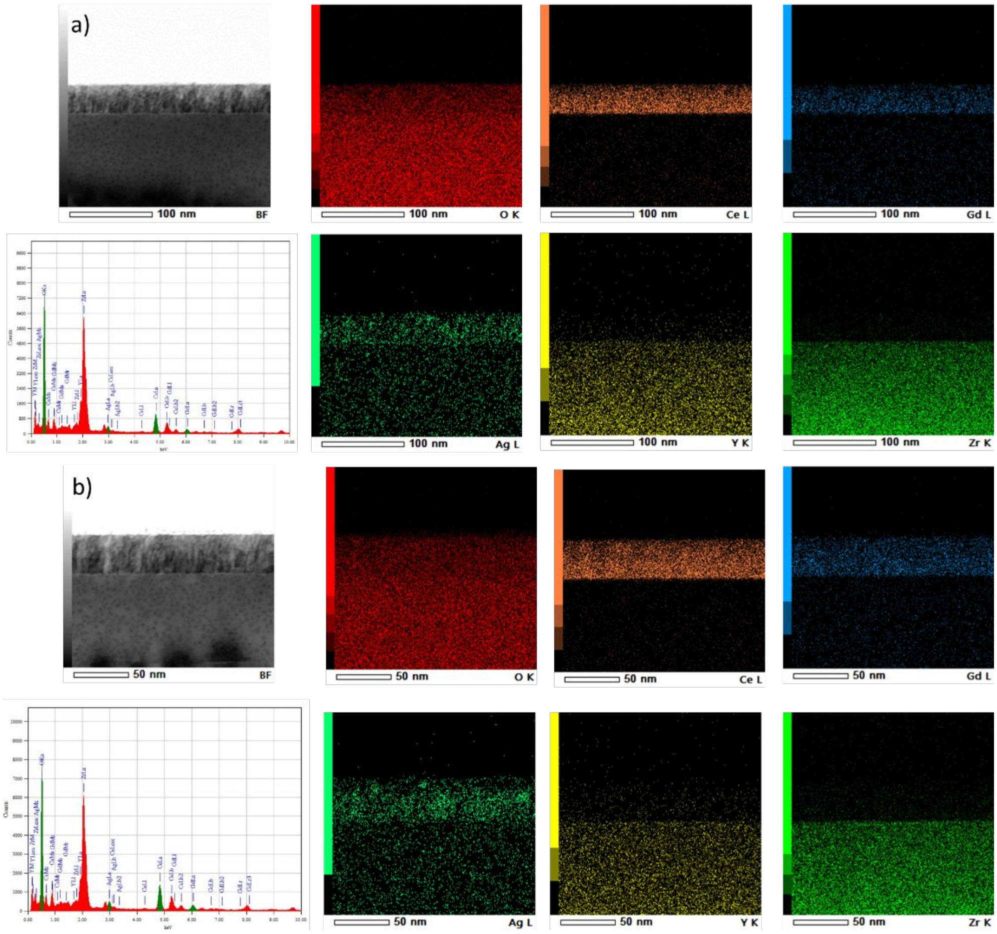

Fig. S2.) Bright field STEM-EDX elemental maps of the ~30 nm thick Ag/CGO film deposited on YSZ substrate for two different zones shown in a) and b). The maps show Ag L edge, Ce L edge, Gd L edge, Zr K edge, Y K edge, and O K edge.

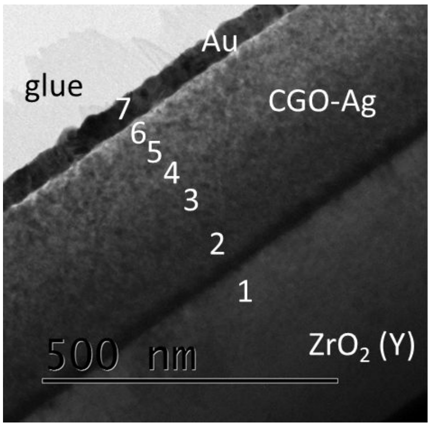

Fig. S3.) Cross-sectional bright field TEM micrograph of the 300 nm thick Ag-CGO film deposited on YSZ (100) substrate. The numbers 1-7 show the position of EDX point analyses. Results are given below in Table S1

Table S1: Elemental composition of each position (1-7) from STEM-EDX point analysis shown in Fig.S3.

Spectra	% Atom C	% Ato O	% Ato Cr	% Ato Cu	% Ato Y	% Ato Zr	% Ato Ag	% Ato Ce	% Ato Gd	% Ato Au
1	37.64	35.34	0.16	3.30	4.64	18.88				
2	49.32	22.64		5.78	0.48	1.86	7.42	11.16	1.30	
3	37.94	27.80		6.71	0.61	2.30	10.26	12.78	1.55	
4	54.95	21.45		4.64	0.45	1.84	6.53	9.06	1.04	
5	57.45	20.37		4.39	0.57	1.86	6.39	7.89	1.03	
6	66.25	16.83		3.49	0.50	1.94	5.06	5.24	0.65	
7	86.78	2.917	0.20	3.59	0.43	1.57	0.92			3.55

Inverse Fourier transform of the HRTEM image using a mask on the Ag (111) reflection revealed the (111) plans of Ag particles, evidencing the polycrystalline nature of the 300 nm film.

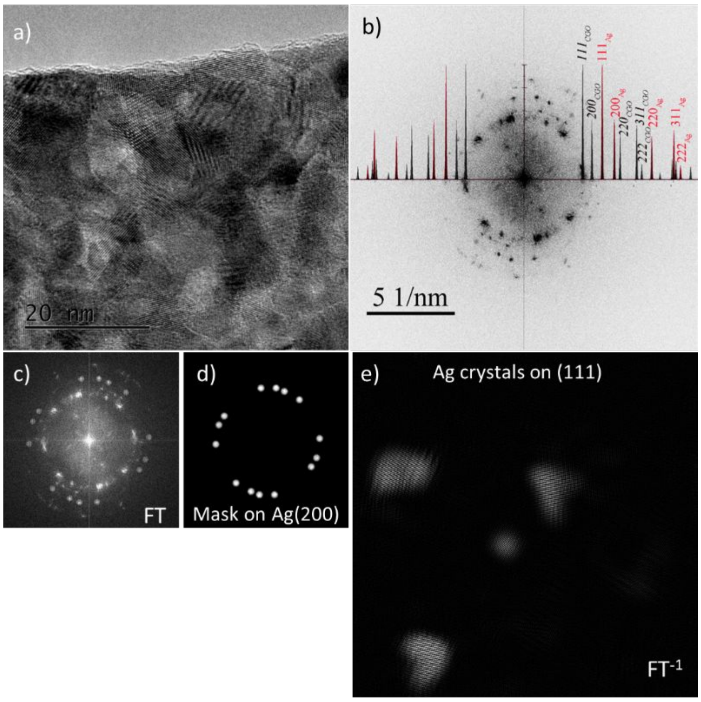

Fig. S4. a) Cross-sectional bright field HRTEM micrograph of the 300 nm thick Ag-CGO film deposited on YSZ (100) substrate, b) SAED pattern of the film, c) and d) show the Fourier transform of image a) and the mask applied on (111) patterns of Ag. e) Inverse Fourier Transform of c) showing the Ag nanoparticles in the (111) orientation.

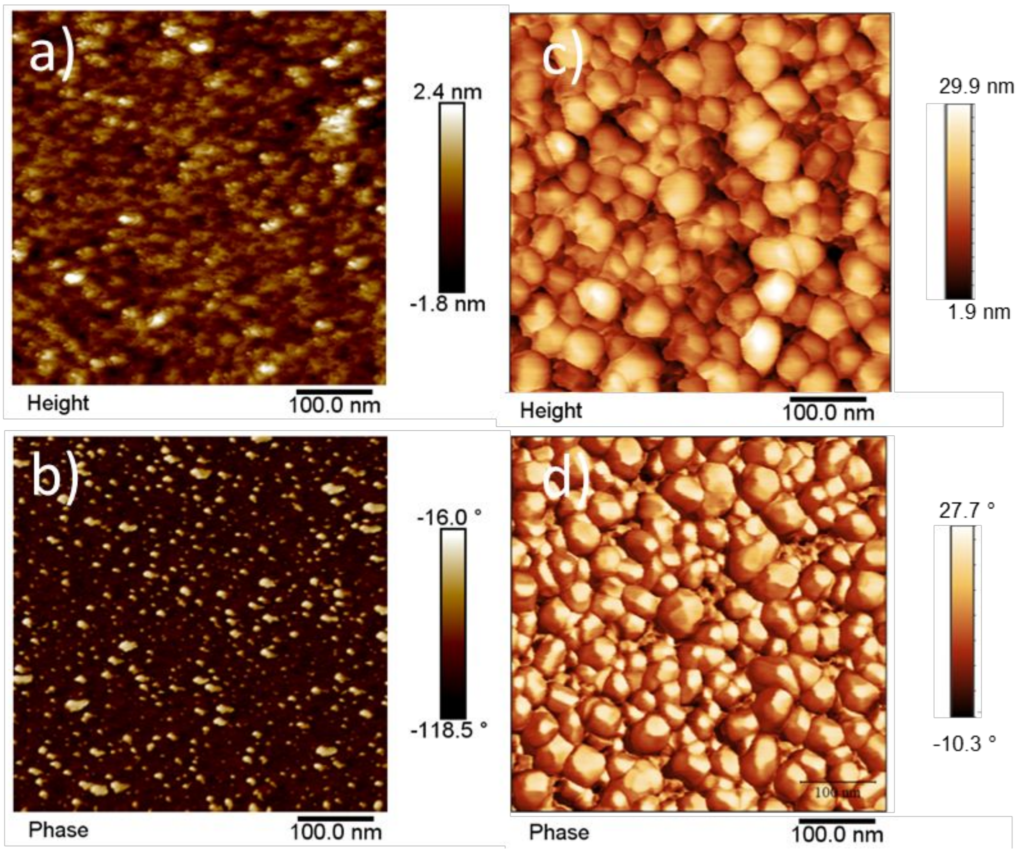

Fig. S5 Atomic force micrographs showing the height and phase difference of the 30 nm in a) and b) respectively, and 300 nm thick film in c) and d), respectively.

The Arrhenius plot of total conductivity measured for the YSZ single crystal electrolyte sandwiched between the thin film electrodes of this study and brush-painted silver film is shown in Fig. S6. In comparison to the reference data from Ahamer et al. [1], it can be seen that the values are slightly lower, while a similar activation energy is achieved. The lower conductivity values in this work might be related to issues with current collection or the real surface area of the electrodes in contact with the electrolyte.

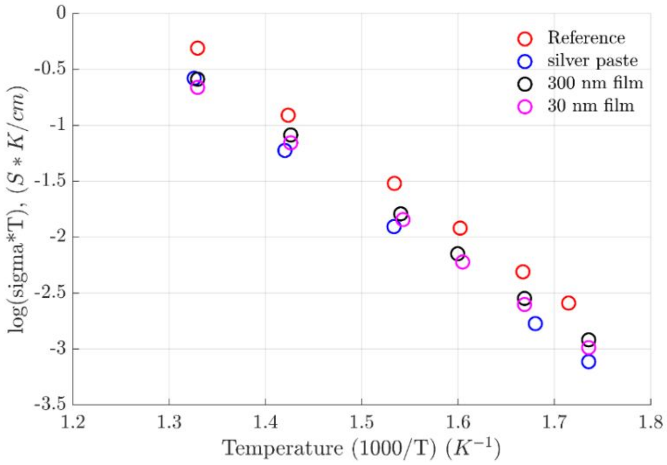

Fig. S6. Arrhenius plot of conductivities measured for the YSZ single crystal. The reference is taken from [1].

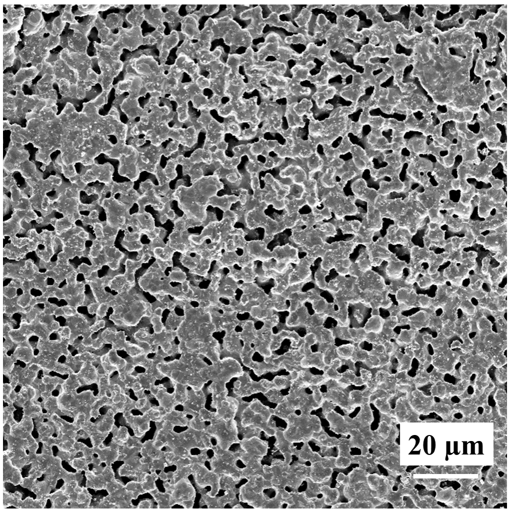

Fig. S7. Top-view scanning electron micrograph of brush-painted Ag electrode.

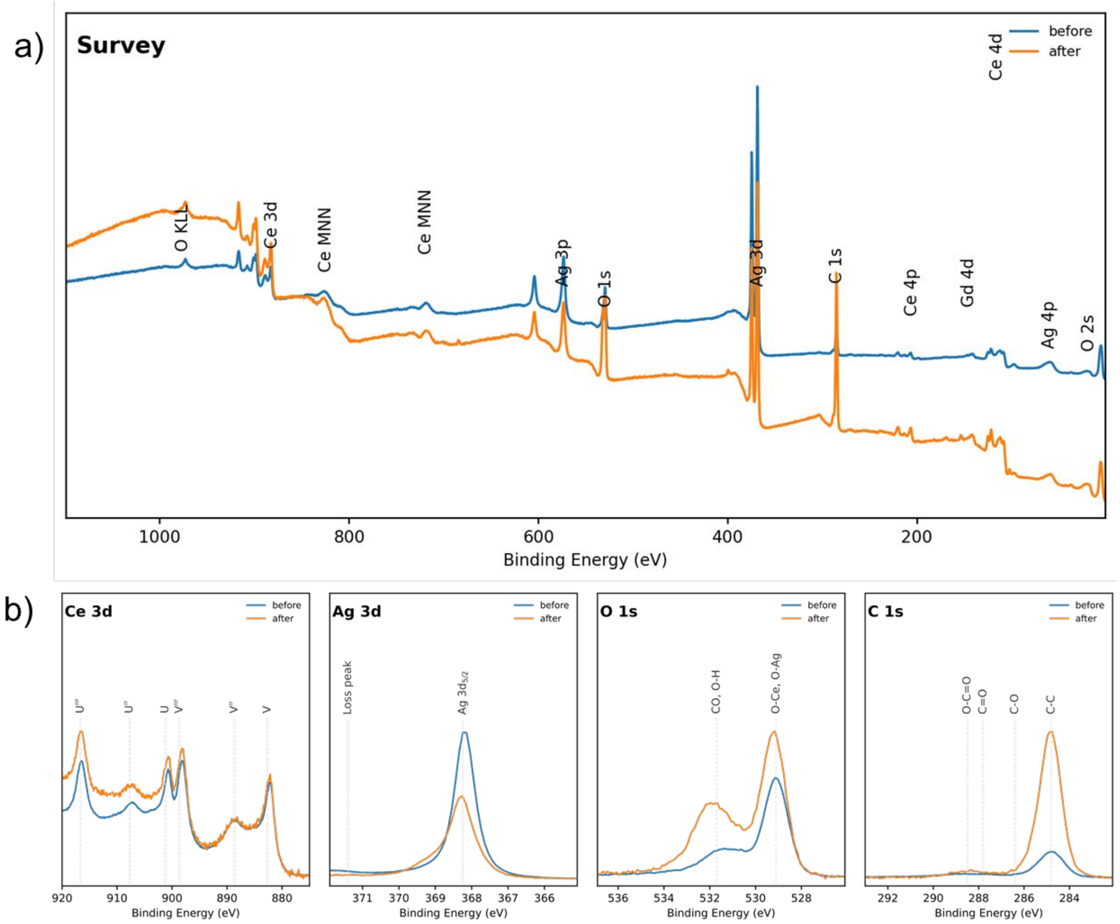

Fig. S8. a) XPS survey spectrum of Ag/CGO thin film before and after stability test. b) Comparison of core level spectra normalized to Ce3d peak before and after stability test.

Comparison of the core-level spectra, normalized to the Ce 3d peak as shown in Fig. S8b, indicates that the apparent reduction in Ag height coinciding with the broadening of the peak and the formation of AgO. Additionally, the O 1s spectrum reveals an increase in the O-Lat contribution, which is attributed to the emergence of O-Ag and O-Ce bonds at approximately 529 eV.

Table S2: XPS fitting results of the pristine sample including % atomic concentration. BE refers to binding energy and FWHM to full width at half maximum. The peak binding energy of the C-C bond is fixed at 284.80 eV.

Name	Peak BE	FWHM eV	Atomic %
Ag3d5/2	368.19	0.68	35.69
Ce3d	882.22	2.44	14.74
Gd4d	141.79	4.44	2.29
O1s O-M	529.09	1.20	29.8
O1s CO,OH	531.26	2.43	17.47

Table S3: XPS fitting results of the aged sample including % atomic concentration. BE refers to binding energy and FWHM to full width at half maximum. The peak binding energy of the C-C bond is fixed at 284.80 eV.

Name	Peak BE	FWHM eV	Atomic %
Ag3d5/2 Ag(0)	368.28	0.62	8.22
Ag3d5/2 Ag(I) AgO	368.35	0.98	6.06
Ag3d5/2 Ag(III)	367.85	0.98	2.28
Ag3d5/2 Ag(III) sat	369.25	0.98	2.59
Ce3d	882.28	2.54	11.16
Gd4d	141.24	4.78	2.73
O1s CO, O-surf	530.47	1.22	4.02
O1s CO,OH, SiO2	531.88	2.00	28.59
O1s O-M	529.20	1.22	34.36

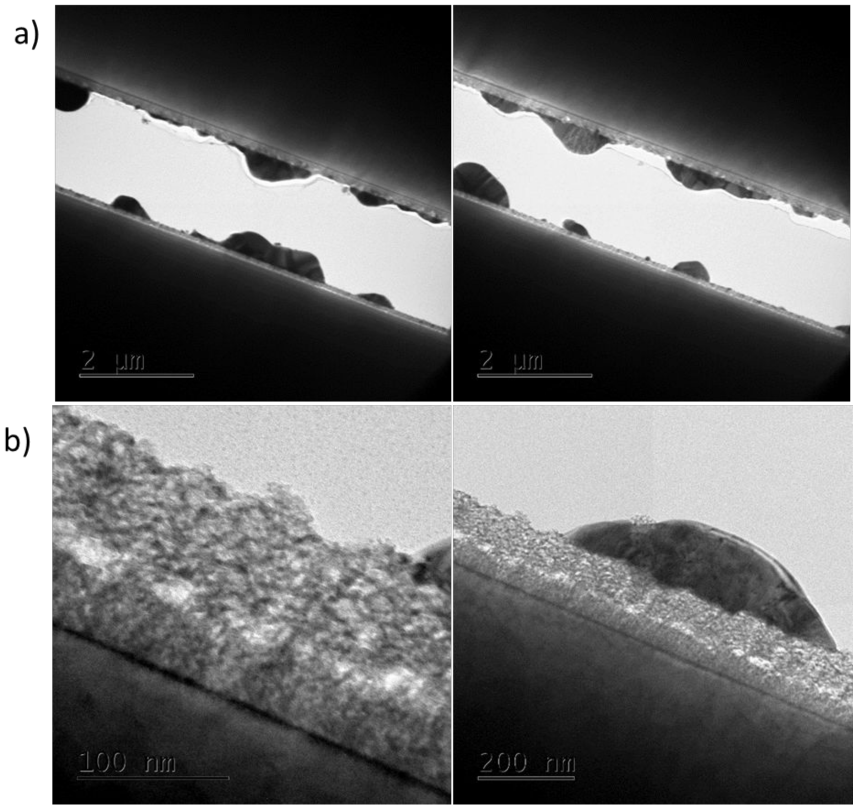

Fig. S9. A) Cross-sectional bright field HRTEM micrograph of the aged 300 nm thick film across 3 different positions, b) showing the film thickness at a higher magnification.

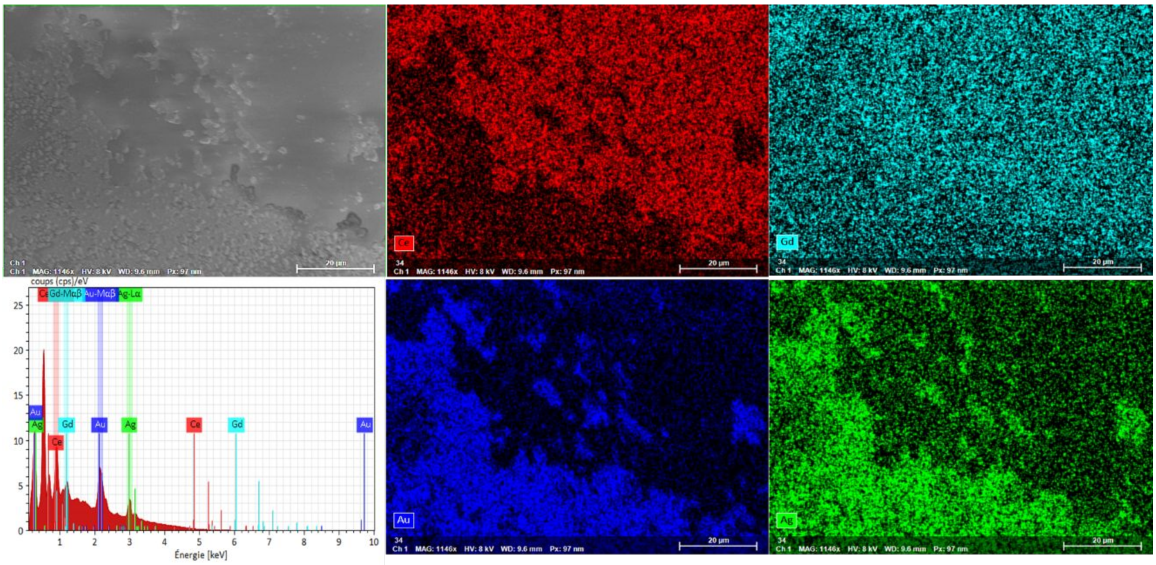

Fig. S10. Top-view scanning electron micrograph of the aged 300 nm film.

References:

[1] Ahamer C, Opitz AK, Rupp GM, Fleig J. Revisiting the Temperature Dependent Ionic Conductivity of Yttria Stabilized Zirconia (YSZ). J Electrochem Soc 2017;164:F790–803.
